# Overexpression of the VRK1 kinase, which is associated with breast cancer, induces a mesenchymal to epithelial transition in mammary epithelial cells

**DOI:** 10.1371/journal.pone.0203397

**Published:** 2018-09-04

**Authors:** Aye M. Mon, A. Craig MacKinnon, Paula Traktman

**Affiliations:** 1 Department of Biochemistry and Molecular Biology, Medical University of South Carolina, Charleston, SC, United States of America; 2 Department of Pathology, Medical College of Wisconsin, Milwaukee, WI, United States of America; 3 Department of Microbiology and Immunology, Medical University of South Carolina, Charleston, SC, United States of America; 4 The Hollings Cancer Center, Medical University of South Carolina, Charleston, SC, United States of America; University of South Alabama Mitchell Cancer Institute, UNITED STATES

## Abstract

Vaccinia-related kinase 1 (VRK1) is a pro-proliferative nuclear kinase. Mice engrafted with VRK1-depleted MDA-MB-231 breast cancer cells have been shown to develop fewer distal metastases than controls, suggesting VRK1 might play a role in cell migration, invasion, and/or colonization. In work described herein, we investigated the impact of VRK1 overexpression on human mammary epithelial cells. In 2D culture, VRK1 overexpression diminishes cell migration and invasion and impairs the migration-associated processes of cell spreading and cytoskeletal rearrangement. VRK1-overexpressing cells show reduced accumulation of the mesenchymal marker vimentin and increased accumulation of the epithelial markers E-cadherin and claudin-1. VRK1 overexpression also leads to reduced levels of the transcriptional repressors snail, slug, and twist1. Cumulatively, these data indicate that VRK1 overexpression augments the epithelial properties of both MCF10a and MDA-MB-231 cells. We further studied the impact of VRK1 on the epithelial properties of MCF10a cells in 3D matrigel culture, in which cells proliferate and form epithelial sheets that mature into hollow spherical acini. VRK1 overexpression significantly accelerates the initial stages of cell proliferation, leading to larger acini that nevertheless differentiate and mature. Our analysis of human tumor tissue microarrays (TMAs) revealed that VRK1 protein levels are higher in lymph node metastases than in patient-matched mammary tumors. Using public databases, we determined that VRK1 is among the top 10% of overexpressed transcripts in multiple subtypes of invasive breast cancer, and that high levels of VRK1 expression are correlated with decreased relapse-free survival. In sum, overexpression of VRK1, by regulating the transcription repressors snail, slug, and twist1, can promote a mesenchymal-to-epithelial transition (MET) in cell culture. VRK1-mediated MET might facilitate the colonization of distal sites by metastatic breast cancer cells, providing some insight into the frequent association of VRK1 overexpression with breast malignancies and the correlation between VRK1 overexpression and poor clinical outcome.

## Introduction

Vaccinia-related kinase 1 (VRK1) is a serine/threonine kinase with a predominantly nuclear localization [1, 2]. It is highly expressed in proliferative tissues, tumors and cancer-derived cell lines [2–9]. VRK1 has been proposed to play a role in cancer progression by promoting the G1 to S cell cycle transition, in part by phosphorylating the CREB1 transcription factor and increasing Cyclin D1 mRNA levels [10, 11]. We have shown that stable depletion of VRK1 in MCF10A and MDA-MB-231 cells (normal and malignant mammary epithelial cells, respectively) significantly slows cell proliferation *in vitro* [5]. VRK1 has also been implicated in the DNA damage response (DDR) induced by UV-light or ionizing radiation. Reports that VRK1 phosphorylates 53BP1 [12], NBS1 [13] and histone H2AX [14] suggest that it may increase tumor resistance to DNA damage-based therapies [6]. Other identified substrates of VRK1 include stress-responsive transcription factors such as p53 [15, 16], cJun [17], and ATF2 [18].

VRK1 phosphorylates the BAF protein (barrier-to-autointegration factor) in early mitosis, and facilitates chromosome condensation and nuclear envelope breakdown [19, 20]. Depletion of VRK1 leads to lagging chromosomes and anaphase bridges, and by causing the aberrant, constitutive association of BAF with mitotic chromosomes, jeopardizes chromosome condensation and nuclear envelope reassembly [20–22]. Mice with hypomorphic expression of VRK1 are infertile due to a loss of proliferating spermatogonia and defects in the ability of oocytes to undergo successful fertilization [23–25], underscoring a crucial role for VRK1 in cell division.

The proliferation and differentiation of normal mammary epithelial cells has often been studied in 3D culture, in which polarized epithelial cells proliferate and then mature to form hollow acini [26]. VRK1 was identified as a protein whose expression is turned off in post-proliferative mature acini, prompting the hypothesis that elevated VRK1 expression might be associated with breast cancer progression [27]. Indeed, an association of VRK1 overexpression with poor clinical outcome was observed in breast cancer patients with ER+ tumors of the luminal A subtype [28, 29]. Our laboratory reported that mice engrafted with VRK1-depleted MDA-MB-231 cells develop smaller primary tumors and fewer distal metastases to the lung and brain [5]. These studies indicate that VRK1 depletion affects both mammary epithelial cell proliferation and tumor metastasis.

To gain further insight into how VRK1 might contribute to breast cancer progression, we sought to determine how VRK1 overexpression might alter the cell biology of mammary epithelial cells. Data described herein show that VRK1 overexpression augments the epithelial properties of MCF10A cells, increasing epithelial cell-cell adhesion, retarding cell spreading, migration and invasion in 2D culture, and promoting cell proliferation in 3D culture. In addition, VRK1 overexpression is associated with reduced levels of the epithelial-to-mesenchymal (EMT)-promoting transcription factors snail, slug, and twist1. Strikingly, analysis of breast tissue microarrays (TMAs) indicates that VRK1 expression is higher in lymph node metastases than in patient-matched primary mammary tumors. VRK1 overexpression may facilitate breast cancer progression by enhancing the mesenchymal-to-epithelial (MET) transition thought to promote cancer cell colonization during metastatic spread [30–35].

## Materials and methods

### Materials

Hygromycin, puromycin, zeocin, collagen, fibronectin, and laminin substrates were purchased from Life Technologies. Matrigel was obtained from Corning. Transwell inserts (CLS3422) were purchased from Sigma. Antibodies used in our studies were: Vimentin (1:4000 WB; 1:500 IF; Abcam), E-Cadherin (1:250 IF in 2D culture; 1:50 IF in 3D culture; BD Biosciences), Snail (1:500 WB; Cell Signaling), Slug (1:500 WB; Cell Signaling), Calnexin (1:1000 WB; Enzo), Bim (1:250 WB; Enzo), laminin V (1:50 IF in 3D culture; EMD Millipore), Claudin1 (1:500 WB; Invitrogen), Twist1 (1:500 WB; Santa Cruz), PCNA (1:250 WB; Santa Cruz), CyclinD (1:250 WB; Santa Cruz), and VRK1 (1:1000 WB; Sigma).

### Cell lines

Human mammary epithelial cells (MCF10A and MDA-MB-231) were obtained from ATCC (Rockville, MD) and cultured as previously described [5]. For human VRK1 overexpression, we used the pHAGE lentiviral system [36, 37] (a generous gift from Dr. Amy Hudson, Medical College of Wisconsin) to generate stable MCF10a (or MDA-MB-231) cells carrying pHAGE vector (pHM), pHAGE-3XFLAG-VRK1 (pHM-3XF-VRK1) or pHAGE-3XFLAG-kinase dead VRK1 (pHM-3XF- VRK1^D177A^) [5]. To deplete human VRK1, we utilized the pLL3.7 LentiLox system (Addgene) and VRK-targeting shRNAs previously published by our laboratory [5]. Experimental analysis was performed on VRK1-depleted cells from 6–21 days post transduction.

### Migration track assay

Two-well glass chamber slides were pre-coated with 50 μg/ml Poly-D-lysine (30min, 37°C) and coated with 1μm diameter fluorescent microspheres (Thermo Scientific) for 1h at 37°C. Chambers were washed three times in PBS. Cells were then added and incubated for 24h at 37°C. Images of clear track areas were taken at 5 random fields/sample at 20X magnification on a Nikon Eclipse Ti microscope.

### Wound scratch assay

Stably-transduced MCF-10A cells were grown to confluency in 35mm dishes for 18h. Confluent monolayers were scratched using a 200μl pipette tip and washed three times with medium. Live imaging was performed on four fields along the wound edge every 30min for 18h using a Nikon Eclipse Ti microscope equipped with Perfect Focus and a Tokai Hit INU Stage Top Incubator (Fujinomiya-shi, Shizuoka-ken, Japan). Wound distances were measured using Nikon NIS software.

### IncuCyte wound scratch assay

Cells were plated in triplicate in 96-well plates and grown to confluency for 18h. Monolayers were wounded using the Incucyte 96-well wound maker; live images were captured at two fields/well every 2h for 14h. Linear regression analysis was used to obtain the slope of the percent wound closure.

### Single cell spreading assay

Cells were seeded on two-well glass chamber slides and incubated at 37°C for the times indicated prior to being fixed and stained with Rhodamine-conjugated phalloidin (to visualize cell area) and counterstained with DAPI. Images from five fields/chamber were taken using a 20X objective on a Nikon Eclipse Ti microscope; cell areas were measured using Nikon NIS software.

### Live imaging of MCF-10A cells expressing GFP-LifeAct

MCF-10A cells that constitutively carry both pHAGE-Zeocin-EGFP-LifeAct [mEGFP-LifeAct-7, Addgene plasmid # 54610)] and either pHAGE-Hygromycin-3XF-VRK1 or empty vector were generated via lentiviral transduction. These dually transduced cells were selected by growth in complete medium supplemented with Zeocin (100μg/ml) and Hygromycin (75μg/ml). Monolayers of confluent cells were wounded and monitored as above. The number of filopodia was scored in a blinded manner.

### Transwell invasion assay

Upper transwell chambers were coated with matrigel (30μg/ml) diluted in serum-free DMEM medium for 30min at 37°C. Cells were serum starved for 18h, trypsinized and added to the upper chamber of the transwells in serum-free DMEM medium. Complete medium supplemented with 10% FCS was added to the bottom chamber. Cells were incubated at 37°C for 16h. Non-invading cells were removed from the upper chamber; invaded cells retained on the bottom surface of the filter were fixed with 4% PFA, and stained with DAPI. Images were taken of four random fields/transwell at 10X magnification on a Nikon Eclipse Ti microscope. Nuclei were counted manually.

### 3D acinus formation assay

3D matrigel cultures [38] were set up in 12-well dishes coated with 400μl matrigel/well. At days 4, 5.5, 7, and 12, brightfield images of acini were captured at 40X magnification on a Nikon Ti microscope for measurement of the acinar areas. In parallel experiments, cells were pulsed with BrdU (25 μg/ml; Sigma) for 1h at 37°C and then harvested using dispase (1mg/ml). Single cell suspensions were stained with FITC-conjugated anti-BrdU (BioLegend) and propidium iodide (Sigma). Samples, along with negative controls (unstained samples, and cells without BrdU), were subjected to flow analysis. Flow data were collected using a Beckman Coulter flow cytometer (Pasadena, CA), CyAn ADP equipped with acquisition software “Summit 4.4”. For immunoblot analysis, acini were released with dispase and immunoblotting was performed as previously described [5].

### Confocal immunofluorescence

Cells were seeded in 3D culture as previously published [38]. Images of acini were taken in z-series at a 20X magnification using the Leica SP5 confocal microscope (Wetzlar, Germany).

### Quantitative real-time PCR

Total RNA was extracted using the RNeasy Mini Kit (Qiagen) and genomic DNA removed using the TURBO DNA free TM kit (ThermoFisher/Ambion). First strand cDNA was synthesized using M-MLV Reverse Transcriptase (ThermoFisher/Ambion). Quantitative real-time PCR was performed (3 biological replicates, each assayed in technical triplicate) on a BioRad CFX384 real-time PCR machine using Power SYBR Green Master Mix (ThermoFisher/Applied Biosystems). RNA fold change was calculated using the ΔΔCT method. Primers are shown in S1 Table.

### Computational studies

Oncomine (www.oncomine.org) was used to compare the levels of the VRK1 transcript in breast cancer vs. normal tissue using the complete Curtis Breast dataset. The search criterion was for VRK1 ranking within the top 10% of overexpressed genes. Kaplan-Meier analyses were generated using the public website: http://kmplot.com/analysis. The Affymetric ID 203856_at was used for VRK1. Plots were generated using relapse-free survival (RFS) as the criterion with 10 years as the follow-up threshold. Statistics were calculated by the Oncomine and Kaplan-Meier Plotter sites.

### Statistical analysis and figure preparation

A Student’s paired two-way *t-test* or Mann-Whitney Rank Sum test was performed to determine the statistical significance of the data obtained from the cell biological experiments. Data were graphed using SigmaPlot (SPSS). Figures were assembled using Canvas software.

### Tumor tissue microarray

Seven human tumor tissue microarrays (TMA) constructed from archival samples were examined, with each case represented by 2–4 tissue cores. The samples analyzed included 44 primary tumors (32 invasive ductal carcinoma, 10 invasive ductal and in situ ductal carcinoma, 2 invasive lobular carcinoma) and 54 lymph nodes. Patient-matched primary tumor and lymph node samples were available for 28 of these cases. Histologically normal breast tissue controls were present in each array. All protocols were approved by the Medical College of Wisconsin (Milwaukee, WI) Institutional Review Board.

### Immunohistochemical (IHC) analysis

IHC was performed using an automated DAKO AutostainerPlus stainer as previously described [39]. The automated cellular imaging system (ACIS) measures the staining intensity based on three parameters: hue, luminosity and saturation. The color-specific thresholds were set by an experienced pathologist and used to calculate both staining intensity and the ratio of positively stained cells to the entire area of selection.

## Results

### Overexpression of VRK1 significantly delays single cell migration

**Fig 1:**
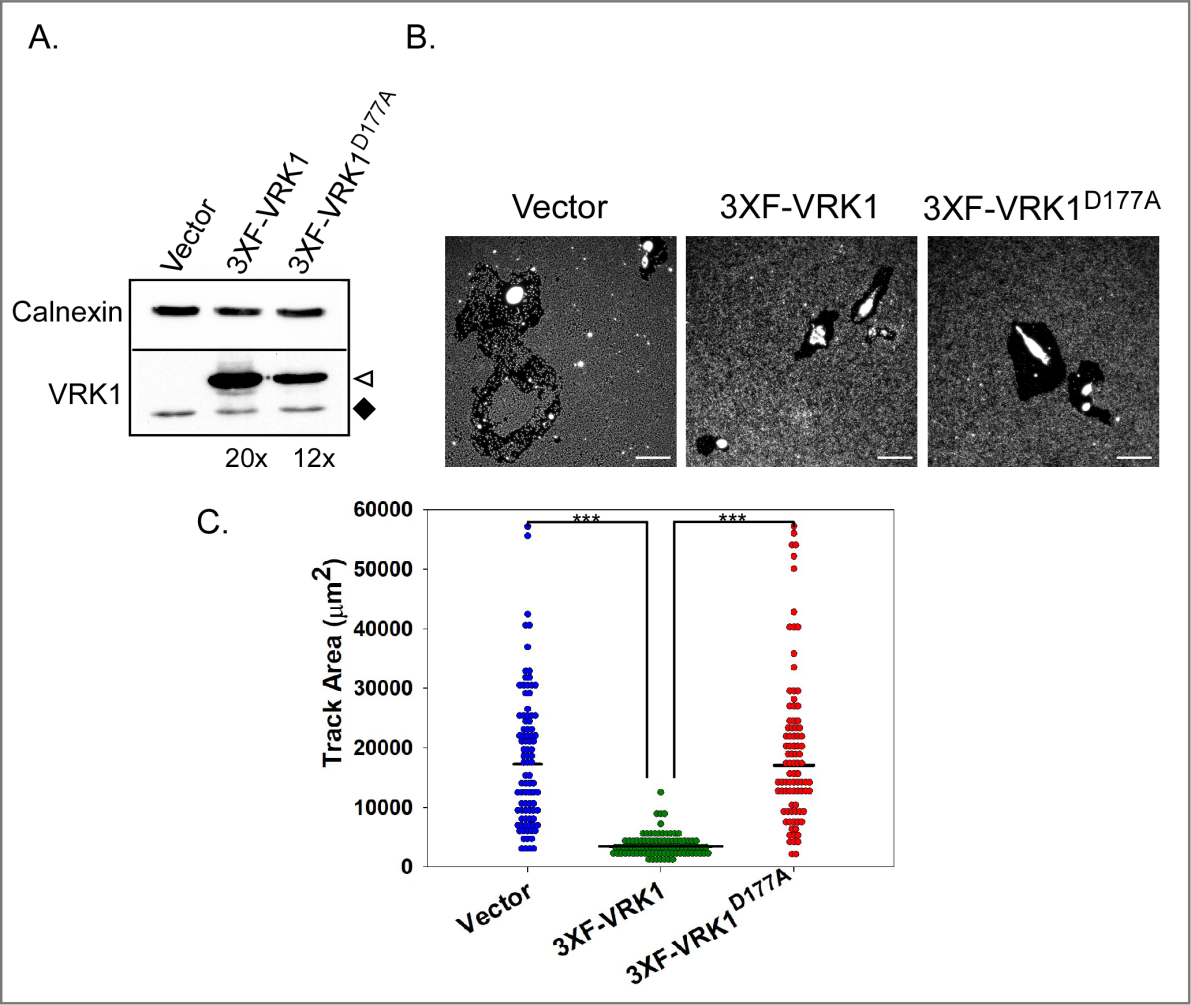
Constitutive overexpression of VRK1 significantly diminishes single cell phagokinetic migration. **(A)** Generation of MCF10A derivatives: MCF10A cells were transduced to stably overexpress 3XF-VRK1 or 3XF-VRK1^D177A^ (kinase-dead allele). Immunoblot analysis was performed to assess protein expression: ◆ highlights endogenous VRK1, **◁** indicates 3XFLAG-VRK1. Levels of 3XF-VRK1 and 3XF-VRK1^D177A^ were estimated to be approximately 20- and 12-fold higher than endogenous VRK1, respectively. **(B)** Representative images of clear tracks generated after 24h of single cell migration are shown. **(C)** Quantitation of the clear track areas generated by the migrating single cells: vector control, 3XF-VRK1-overexpressing and 3XF-VRK1^D177A^-overerxpressing cells are shown with blue, green and red circles, respectively. Bars indicate the median; statistical significance was determined by Mann-Whitney test; *** p<0.001 (n = 90 cells).

Our demonstration that mice engrafted with VRK1-depleted MDA-MB-231 cells developed fewer distal metastases than mice engrafted with control cells [5] suggested that VRK1 might modulate cell migration, invasion, or metastatic colonization. To examine the impact of VRK1 overexpression on the properties of mammary epithelial cells, we chose to focus on MCF10a cells because they are non-transformed, maintain a near-diploid karyotype and have the ability to form polarized acini in 3D culture. We generated MCF10a cells constitutively expressing 3XF-VRK1 or a kinase-dead variant, 3XF-VRK1^D177A^ [19]. Control cells were transduced with the corresponding empty lentivirus vector. The WT and kinase-dead proteins were expressed at ~20- and 12-fold higher levels than endogenous VRK1, respectively (Fig 1A); it is important to note that VRK1 overexpression does not affect the apparent doubling time of MCF10a cells in 2D culture [25.9 +/- 0.5h (vector) vs. 24.6 +/-1.5h (VRK1-overexpressing) and [5]].

Cancer cells can utilize both single cell and epithelial sheet migration to achieve motility during cancer progression. We first assessed the impact of VRK1 overexpression on single cell migration by performing a phagokinetic migration assay [40]. As cells migrate, they ingest the beads and form non-fluorescent tracks (Fig 1B and 1C). Surprisingly, the tracks generated by 3XF-VRK1 cells (Fig 1C, green circle) were ~5 times smaller than those generated by control cells or cells expressing the kinase-dead 3XF-VRK1^D177A^ (compare blue and red circles). These data show that overexpression of catalytically active VRK1 significantly impairs single cell migration.

### Overexpression of VRK1 retards epithelial sheet migration

**Fig 2:**
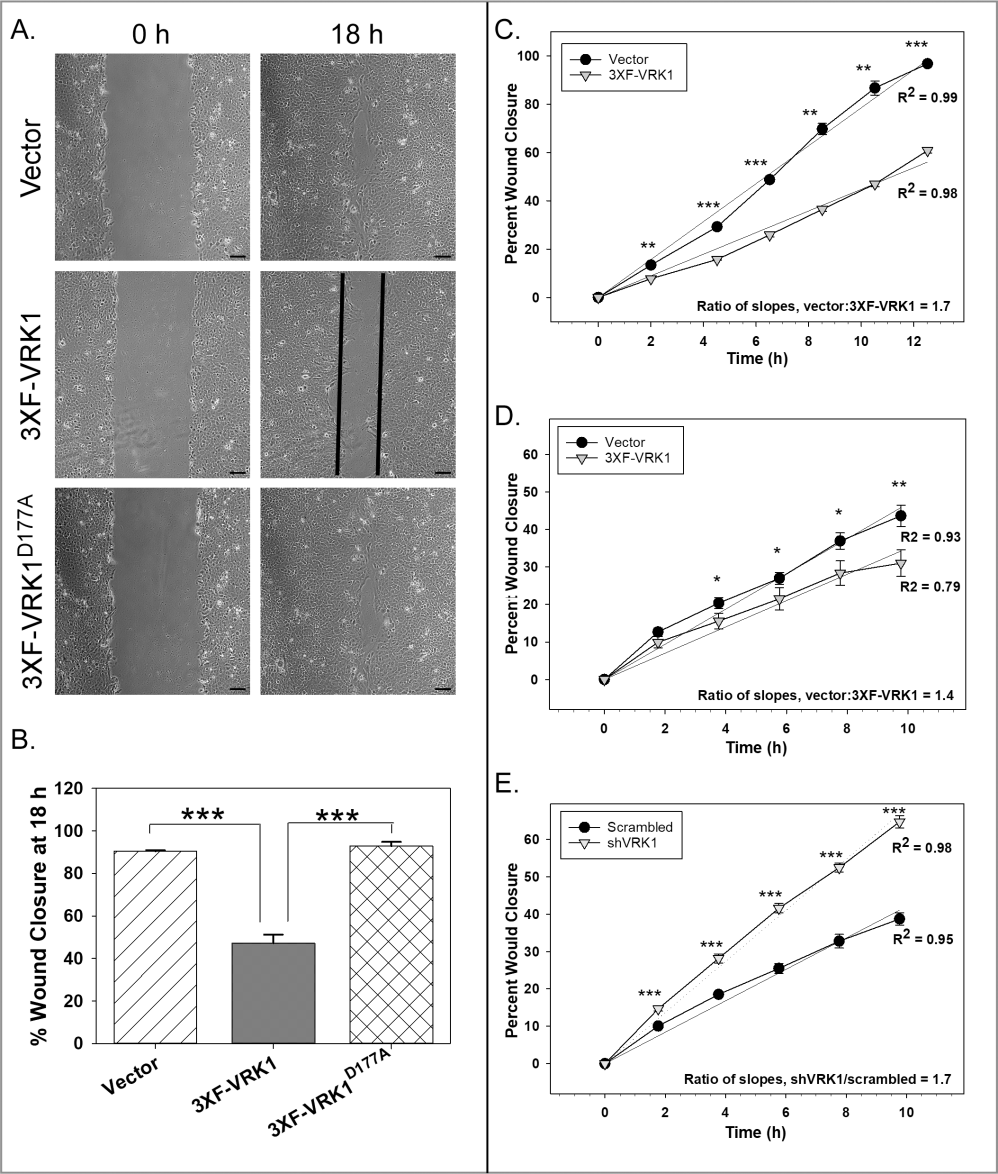
VRK1 overexpression significantly impairs epithelial sheet migration. **(A and B)** Confluent monolayers of the cell lines shown were wounded, and wound closure was monitored for 18h by live imaging microscopy. **A:** Representative images of vector control, 3XF-VRK1-overexpressing and 3XF-VRK1^D177A^-overexpressing cells are shown at 0 and 18h after wounding. Scale bar = 100μm. **B:** Quantification of the percent wound closure at 18 h (Mean and standard error are shown; ***p<0.001) (n = 4 images) **(C-E)** Data from representative IncuCyte wound scratch assays are shown. Using the IncuCyte live imaging system, the rate of wound closure was compared pairwise for vector control MCF10A cells (black circles) and 3XF-VRK1 overexpressing MCF10A cells (gray triangles) **(C)**, or for vector control MDA-MB-231 cells (black circles) vs. 3XF-VRK1- overexpressing MDA-MB-231 cells (gray triangle) **(D)**, or for scrambled shRNA control MDA-MB-231 cells (black circles) vs. VRK1-depleted MDA-MB-231 cells (gray triangles) **(E)**. Slope ratios were determined after linear regression analysis. Significance was assessed using a Student’s *t-test* for each time point (mean and standard error are shown; *p ≤ 0.05; **p ≤ 0.01;***p ≤ 0.001) (n = 6 images).

We also tested the contribution of VRK1 overexpression to epithelial sheet migration by performing wound healing assays (Fig 2A). Confluent monolayers of vector control, 3XF-VRK1 overexpressing and 3XF-VRK1^D177A^ overexpressing MCF10a cells were wounded, and live imaging microscopy was used to monitor wound closure every 30min for 18h. (See S1, S2 and S3 Movies for full videos.) At 18hpi, 3XF-VRK1-overexpressing cells (black) had accomplished only 50% wound closure, while both control (hatched) and 3XF-VRK1^D177A^ (cross-hatched) cells exhibited 90% wound closure (Fig 2B). These data indicate that VRK1 overexpression significantly slows epithelial sheet migration. To measure the rate of wound closure more accurately, we performed IncuCyte wound scratch assays; images were taken every 2h for 13h. Linear regression analysis indicated that 3XF-VRK1-overexpressing MCF-10A cells (gray triangles) migrated ~1.7 times more slowly than control cells (black circles) (Fig 2C).

To determine whether VRK1 depletion, conversely, would accelerate sheet migration, we used MDA-MB-231 cells overexpressing 3XF-VRK1 (~15-fold above endogenous levels) or depleted of VRK1 (>95% depleted) (S1A and S1B Fig), because VRK1-depletion has a more modest impact on the proliferation rate of MDA-MB-231 cells than it does on MCF10a cells [5]. We observed that 3XF-VRK1 overexpressing MDA-MB-231 cells (gray triangles) migrated approximately 1.4 times more slowly than control cells (black circle) (Fig 2D). Conversely, we found that MDA-MB-231 cells depleted of VRK1 (gray triangles) migrated approximately 1.7 times faster than the control cells (black circles) (Fig 2E). In sum, our data strongly support that, in 2D culture, VRK1 overexpression retards epithelial sheet migration, while VRK1 depletion promotes sheet migration.

### VRK1 overexpression does not affect cell:Matrix adhesion but does affect cell spreading

**Fig 3:**
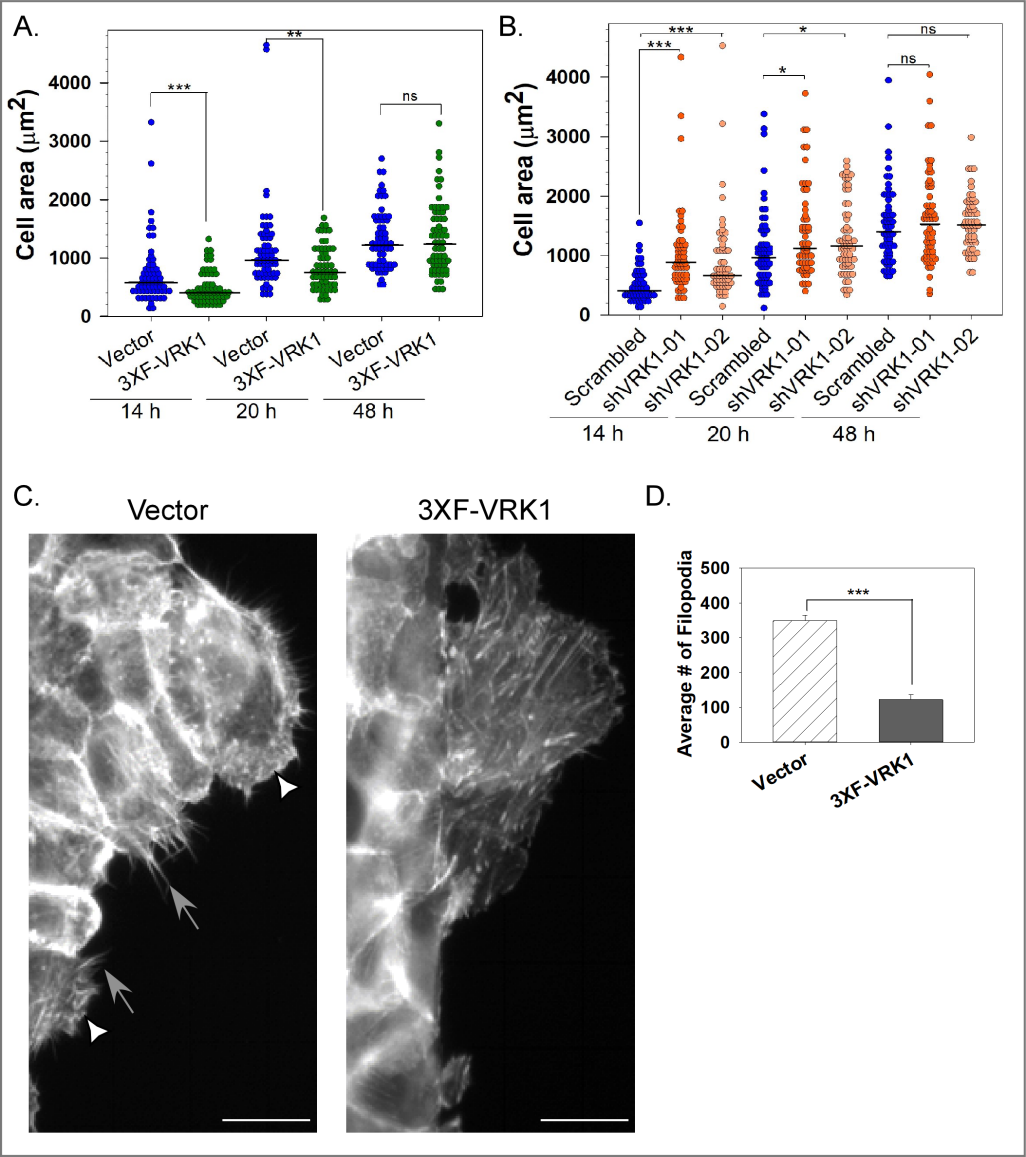
VRK1 levels modulate cell spreading and cytoskeletal rearrangement. **(A)** To assess the rate of cell spreading, the areas of individual vector control (blue circles) and 3XF-VRK1-overexpressing (green circles) MCF10A cells were measured at 14, 20, and 48h post seeding (n = 60 cells). **(B)** As for panel A, the areas of individual scrambled shRNA control (blue circle), sh-VRK1-01 (orange circle), and sh-VRK1-02 (light orange circle) MCF10a cells were measured at 14, 20, and 48h post seeding (n = 60 cells). In Figs A and B, bars indicate the median; statistical significance was determined by Mann-Whitney test. **(C and D)** Stable MCF0a cells co-expressing either GFP-LifeAct and vector control or GFP-LifeAct and 3XF-VRK1, were wounded and monitored for the rearrangement of the cytoskeleton using time-lapse imaging. Representative images of the cytoskeletal organization of cells along the leading edge are shown in **C**. White triangles indicate lamellipodia and gray arrows indicate filopodia. Scale bar = 100 μm. Quantification of the average number of filopodia per 40X magnification field is shown in **D**. (Mean and standard error shown; ***p<0.001) (n = 6 images).

The migration of individual cells on a 2D surface is thought to involve contact of the leading edge with the adhesive surface, contraction of the cell, and then retraction of the lagging edge [41]. To dissect the influence of VRK1 on cell migration, we therefore examined adhesion to dishes left uncoated or coated with various components of the ECM, but found no significant differences between control and VRK1-overexpressing MCF10a cells (S2B and S2C Fig). We then assessed cell spreading, which involves both adhesion and active cytoskeletal rearrangement [42]. At both 14 and 20h post seeding, the median area of VRK1-overexpressing cells (green circles) was ~1.4 times smaller than that of control cells (blue circles) (p<0.001, <0.01, respectively) (Fig 3A). However, at 48h post seeding, when cells have reached their steady state morphology, no significant differences were seen (Fig 3A).

Using the same assay, we compared MCF10A cells stably depleted of VRK1 to control cells (S1C Fig). At 14 and 20h post seeding, the median area of cells stably depleted of VRK1 (shVRK1-01, orange and shVRK1-02, light orange) was 2.0 and 1.8 times larger than that of scrambled cells (blue), respectively (***p<0.001, *p<0.05) (Fig 3B). Together, the data indicate that VRK1 overexpression significantly delays cell spreading while VRK1 depletion accelerates cell spreading.

During cell spreading and migration, actin-rich protrusive structures such as filopodia and lamellipodia sense the extracellular environment. After performing a wound-scratch assay on MCF10A cells engineered to co-express GFP-LifeAct, we observed a significant reduction (3-fold) in the number of filopodia in 3XF-VRK1 overexpressing cells (Fig 3C and 3D, black) relative to control cells (hatched) in our live imaging analysis. No significant difference in lamellipodia formation was seen (data not shown). These data suggest that defects in cytoskeletal rearrangement may underlie the cell spreading and migration phenotypes observed in VRK1-overexpressing cells.

### VRK1 overexpression impedes cell invasion

Because VRK1 overexpression significantly impairs cell migration, we determined the effect of VRK1 overexpression on transwell cell invasion in both MCF10A and MDA-MB-231 cells. The number of 3XF-VRK1 overexpressing MCF10A (black) and MDA-MB-231 (black) cells that had invaded was reduced to 32% ± 2.1 and 22% ± 2.4 of the control MCF10A (hatched) and MDA-MB-231 (hatched) cells, respectively (S3A and S3B Fig) (***p<0.001) (n = 3).

### VRK1 regulates the expression of epithelial and mesenchymal markers

**Fig 4:**
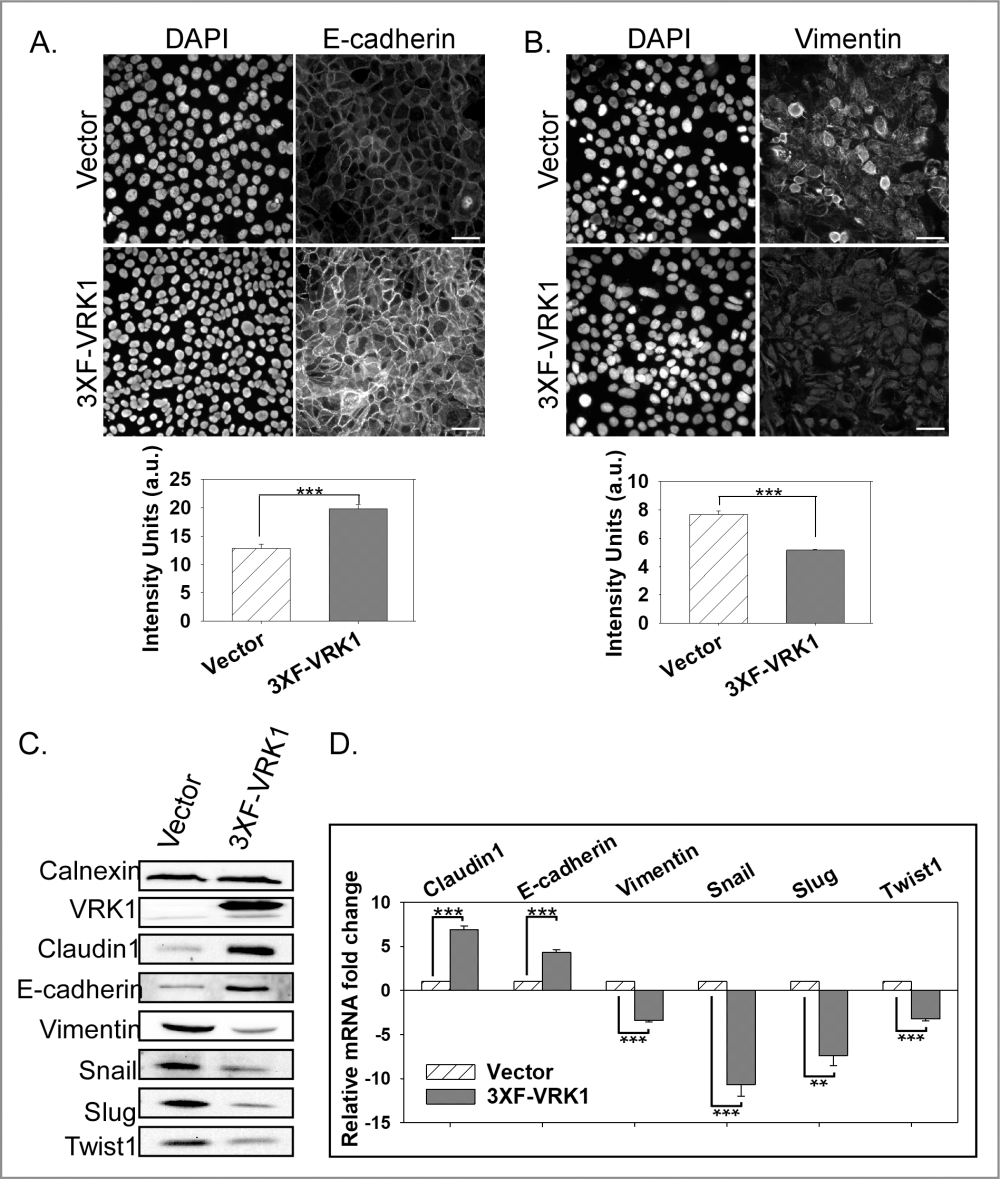
VRK1 overexpression augments the epithelial properties of MCF10A cells. **(A and B)** Immunofluorescence analysis of E-Cadherin (A) and vimentin (B) in control and 3XF-VRK1-overexpressing MCF10a cells. Representative images of E-cadherin and vimentin staining are shown in top panels. Scale bar = 100μm. Quantitation of the E-cadherin and vimentin staining intensity from 6 different random fields is shown in bottom panel (mean and standard error shown; ***p<0.001). **(C)** Immunoblot analysis of control and 3XF-VRK1 overexpressing MCF10A cells. A representative immunoblot is shown (n = 4). **(D)** Quantification of the levels of claudin-1, E-cadherin, vimentin, snail, slug, and twist1 mRNAs by qRT-PCR analysis. Grouped bar graph displays the mRNA fold changes observed in VRK1-overexpressing vs. control cells (mean and standard error shown; **p<0.01, and ***p<0.001) (n = 3).

The cumulative data described above are consistent with VRK1 overexpression augmenting the epithelial phenotype. We therefore examined the levels of epithelial markers by immunofluorescence and immunoblot analysis. E-cadherin and the mesenchymal marker vimentin were visualized by immunofluorescence analysis (Fig 4A and 4B, respectively); quantification of the images indicated that VRK1-overexpressing cells (black) exhibit 1.5-fold higher levels of E-cadherin staining and 1.5-fold lower levels of vimentin staining than control cells (hatched). Complementary immunoblot analyses revealed that the steady-state levels of both E-cadherin (2.5 ± 0.4 fold) and the tight-junction marker claudin-1 (2.0 ± 0.5 fold) were increased in VRK1-overexpressing cells (Fig 4C). Conversely, VRK1-overexpressing cells have reduced levels of vimentin (3.6 ± 0.3 fold). We also assessed the steady-state levels of claudin-1, E-cadherin, and vimentin mRNAs (Fig 4D). mRNA levels for claudin-1 and E-cadherin were significantly higher in VRK1-overexpressing (black) vs. control (hatched) cells (6.9 ± 0.4 and 4.3 ± 0.3 fold, respectively); vimentin mRNA, conversely, was reduced (3.4 ± 0.2 fold). These data provide a plausible molecular basis for the enhanced epithelial behavior of VRK1-overexpressing MCF10A cells.

**Fig 5:**
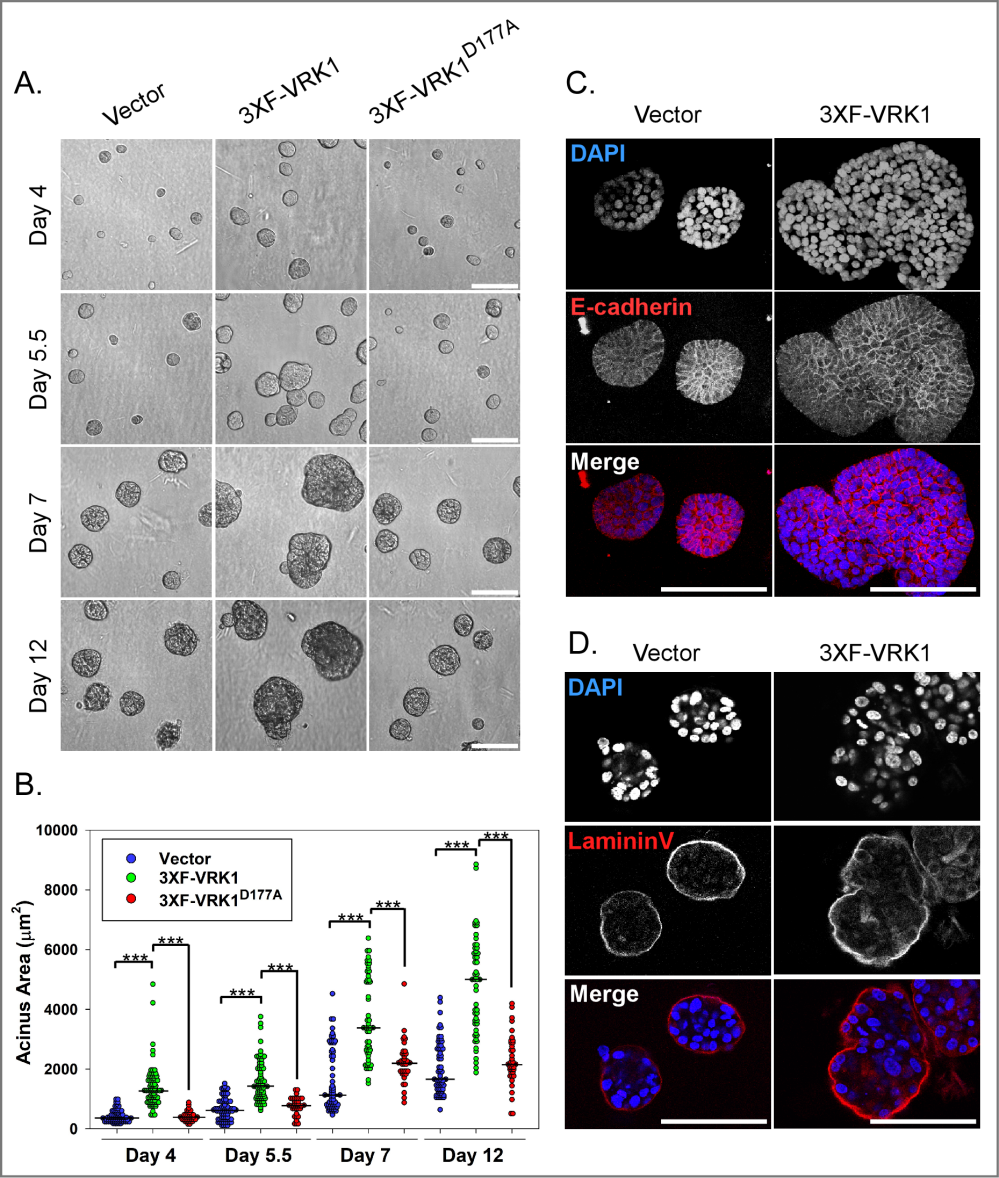
VRK1-overexpression leads to the formation of enlarged and misshapen acini in 3D matrigel culture. **(A)** Representative brightfield images of 3D acini formed by control, 3XF-VRK1- overexpressing cells and 3XF- VRK1^D177A^-overexpressing MCF10a cells at days 4, 5.5, 7, and 12 of 3D culture. Scale bar = 100μm. **(B)** Quantification of the acinus areas at days 4, 5.5, 7, and 12 of 3D culture [n = ~70, vector (blue circles) or 3XF-VRK1 (green circles); n = ~35, 3XF-VRK1^D177A^ (red circles)]. Bars indicate the median. (Mann-Whitney test, ***p<0.001). **(C and D)** Confocal immunofluorescence analysis of 3D acini formed by control and 3XF-VRK1 overexpressing MCF10a cells after 7 days of culture. Acini were stained for the epithelial cell-cell junction marker E-cadherin **(C)** and basal polarity marker laminin V **(D)**. Scale bar = 100μm.

To take this analysis further, we assessed the levels of the transcriptional repressors snail, slug and twist1, which directly regulate the expression of E-cadherin and claudin-1. Strikingly, VRK1 overexpression leads to a significant decrease in the steady-state levels of the snail (5.8 ± 3.1 fold), slug (4.4 ± 0.8 fold), and twist1 (3.3 ± 1.6 fold) proteins (Fig 4C). This decrease was explained by reduced levels of the snail, slug, and twist1 mRNAs in VRK1-overexpressing cells (compare black to hatched, Fig 5D) by 10.7 ± 1.3, 7.3 ± 1.1 and 3.2 ± 0.2 fold, respectively.

Cumulatively, these data establish that VRK1 overexpression promotes increased accumulation of the epithelial markers E-cadherin and claudin-1, while causing decreased accumulation of the EMT-transcriptional repressors snail, slug, and twist1 and the mesenchymal marker vimentin, consistent with a partial mesenchymal-to-epithelial transition (MET).

### VRK1 overexpression leads to the formation of enlarged and misshapen acini in 3D culture

Because VRK1 overexpression augments the epithelial properties of MCF10A cells, we addressed the impact of VRK1 overexpression on the growth of MCF10A acini in 3D matrigel culture, in which cells first proliferate and form spherical epithelial sheets, known as acini. Cell proliferation gives way to differentiation, and eventually interior cells not in contact with the extracellular matrix undergo anoikis, yielding acini with hollow lumens [38]. An initial morphological assessment of the acini at days 4, 5.5, 7, and 12 days after seeding revealed that 3XF-VRK1 cells (green) formed acini whose median size was 1.5–3.6 times larger than those formed by control (blue) or 3XF-VRK1^D177A^ (red) cells (Fig 5A and 5B). 3XF-VRK1 overexpressing acini were also distorted in shape, which is reflected in the bigger spread of the data points in the dot density plot (Fig 5B).

When acini were immunostained for the basal polarity marker laminin V and the cell-cell junction marker E-cadherin at day 7 post-seeding, no disruption in cell polarity or cell:cell junction formation was seen in 3XF-VRK1 overexpressing acini (Fig 5C and 5D). Together these findings clearly indicate that VRK1 overexpression accelerates acinus enlargement and promotes aberrant acinar shape in 3D culture without compromising the integrity of the polarized epithelium.

### Constitutive VRK1 overexpression accelerates cell proliferation during acinus formation

**Fig 6:**
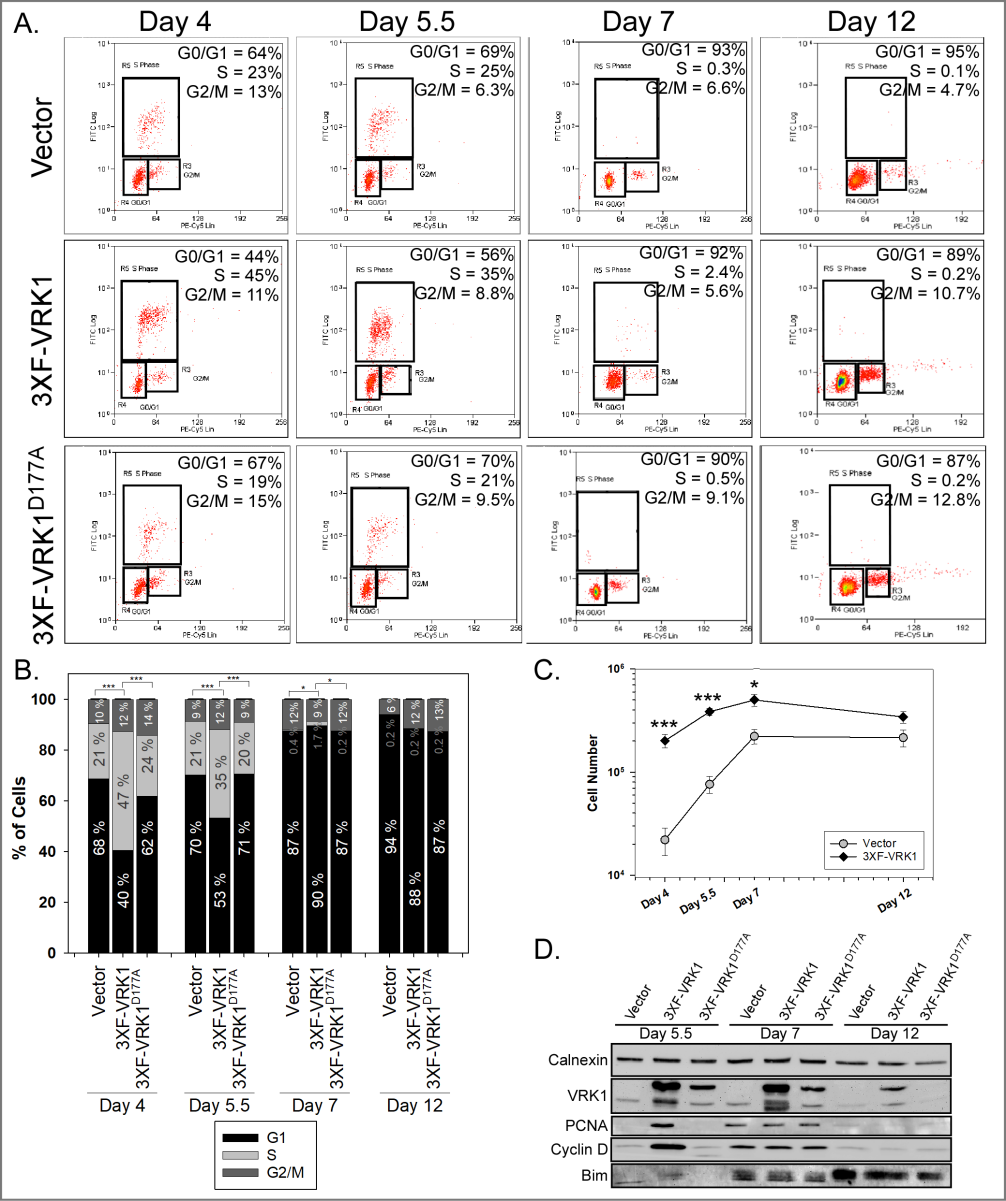
VRK1 overexpression in MCF-10A cells augments cell proliferation during the initial stages of acinus formation. **(A)** Cell cycle analysis was performed on control, 3XF-VRK1-overexpressing and 3XF-VRK1^D177A^-overexpressing MCF10a acinar cells harvested at days 4, 5.5, 7 and 12 of 3D matrigel culture. BrdU-pulsed cells were stained with anti-BrdU and propidium iodide and analyzed by flow cytometry. Representative flow data is shown, with the percentage of cells in G0/G1, S and G2/M shown in the top right of each panel. **(B)** Quantification of the percentage of cells that were in G0/G1 (black bar), S (gray bar), and G2/M (dark gray bar) at different time points (mean and standard error shown; *p <0.05; ***p < 0.001) (n = 3). **(C)** Plot of the total cell number in control or 3XF-VRK1-overexpressing acini harvested at days 4, 5.5, 7 and 12 of 3D culture (mean and standard error shown; *p<0.05, ***p<0.001) (n = 3). **(D)** Immunoblot analysis of control, 3XF-VRK1-overexpressing and 3XF-VRK1^D177A^-overexpressing MCF10a acinar cells harvested at days 5.5, 7, and 12 of 3D culture. Representative blots are shown (n = 2).

The impact of VRK1 on acinus growth could reflect enhanced proliferation or diminished apoptosis. We therefore pulsed cells at days 4, 5.5, 7, and 12 of 3D culture with BrdU for 1h before harvesting the acini and subjecting them to analysis by flow cytometry. In the day 4 and 5.5 acini formed by 3XF-VRK1 overexpressing cells, the percent of cells in S-phase was approximately 2-fold greater than that observed in the acini formed by control or 3XF- VRK1^D177A^ cells (Fig 6A). This increase in the S-phase population in 3XF-VRK1-overexpressing cells was accompanied by a concomitant decrease in the G0/G1-phase population. In the samples harvested at later times (days 7 and 12), the vast majority of the cells were in G0/G1 in all cell lines (Fig 6A and 6B), reflecting the transition from proliferation to differentiation. By counting the total number of cells in the acini before preparing them for flow cytometry, we determined that there were significantly more cells in the VRK1-overexpressing samples (10-fold, 5.3-fold, 2.2-fold, 1.7-fold) than the control samples (black diamonds vs. gray circles) at days 4, 5.5, 7 and 12 of 3D culture (Fig 6C). Cumulatively, our data are consistent with constitutive VRK1-overexpression accelerating an initial burst of cell proliferation.

Lysates of acini harvested at days 5.5, 7 and 12 were subjected to immunoblot analysis for the proliferative markers PCNA and cyclin D and the luminal apoptotic marker, Bim. Consistent with our flow analysis, we observed increased levels of PCNA (~20 fold) and Cyclin D (~12 fold) in VRK1-overexpressing cells at day 5.5, as compared to control or 3XF-VRK1^D177A^ cells. By day 7, the steady-state levels of these two proteins were equivalent in all samples and by day 12, proliferative markers were barely detectable. VRK1 overexpression, therefore, accelerates but does not extend the duration of proliferation in the acini. In addition, the luminal apoptotic marker Bim was observed at similar levels in all of the acini harvested at days 7 and 12, indicating that the onset of apoptosis, diagnostic of acinus maturation, is not affected by VRK1-overexpression (Fig 6D).

### VRK1 overexpression is common in breast cancer, is associated with tumor progression, and has prognostic implications

**Fig 7:**
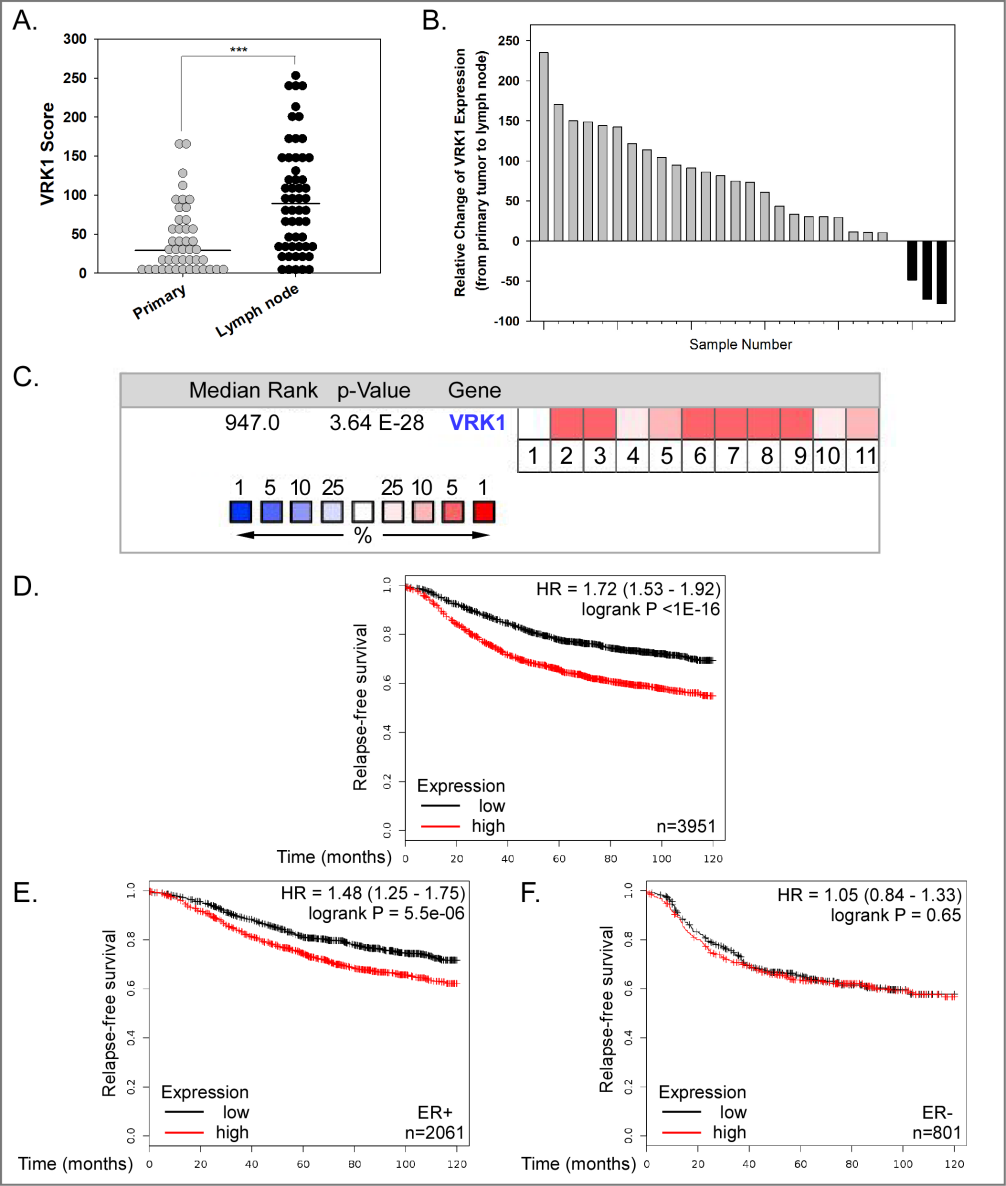
VRK1 overexpression is seen in human breast cancer and is associated with decreased relapse-free survival. **(A)** Immunohistochemical analysis of VRK1 expression in breast tissue microarray. VRK1 score was determined by multiplying the VRK1 staining intensity × percentage of VRK1 positive staining. Fig 8A includes the data from all primary tumor and lymph node samples. Bars indicate the median. (Mann-Whitney test, ***p<0.001). **(B)** VRK1 protein expression is higher in the vast majority of lymph node metastases (24 of 28) than in patient-matched primary tumors. **(C)** Oncomine.org was used to probe the complete Curtis Breast dataset (2,136 samples) in order to determine whether the VRK1 mRNA was within the top 10% of overexpressed transcripts in breast cancer samples vs. normal breast tissue. The overall median rank of VRK1 mRNA overexpression was 947 (out of 19,273 measured genes) (p = 3.64x10^-28^). Data for cancer subtypes are shown in boxes numbered 1–11: 1: benign breast neoplasm (rank 6459, 1.4 fold-change, NS), 2: breast carcinoma (rank 833, 1.5 fold-change, p = 3.83x10^-5^), 3: breast phyllodes tumor (rank 512, 1.2 fold-change, p = 0.002), 4: ductal breast carcinoma in situ (rank 1969, 1.5 fold-change, p = 0.002), 5: invasive breast carcinoma (rank 1060, 1.6 fold-change, p = 1.28x10^-5^), 6: invasive ductal and invasive lobular breast carcinoma (rank 941, 1.5 fold-change, p = 6.76x10^-22^), 7: invasive ductal breast carcinoma (rank 613, 1.5-fold change, p = 2.71x10^-71^), 8: invasive lobular breast carcinoma (rank 947, 1.3 fold-change, p = 3.64x10^-28^), 9: medullary breast carcinoma (rank 400, 1.8 fold-change, p = 3.25x10^-12^), 10: mucinous breast carcinoma (rank 1932, 1.4 fold-change, p = 7.41x10^-10^) and 11: tubular breast carcinoma (rank 1253, 1.4 fold-change, p = 1.24x10^-17^). The median rank of overexpression is shown by the spectrum of white-pink-red colors shown in the legend; VRK1 is in the top 5% of overexpressed mRNAs in subsets 2,3,6–9 and in the top 10% of overexpressed mRNAs in subsets 5 and 11. **(D-F)** Correlation of VRK1 overexpression and decreased relapse-free survival of breast cancer patients. Data analysis using Kaplan-Meier plotter reveal the probability of relapse-free survival for 3,951 breast cancer patients with low (black line) or high (red line) VRK1 expression. **(D)** HR [hazard ratio] of 1.72, p<1x10^-16^). **(E)** Similar analysis of 2,061 patients with ER^+^ breast cancer (HR 1.48, p = 5.5x10^-6^). **(F)** Similar analysis of 801 patients with ER- breast cancer (HR 1.05, p = 0.65). A significant correlation between high VRK1 expression and reduced relapse–free survival is seen for patients with ER+ breast cancer.

Finally, to address the association of VRK1 expression levels with human breast cancer and its progression, we assessed VRK1 protein expression in a custom tumor tissue microarray (TMA). The median level of VRK1 expression was significantly higher in lymph node metastases (black circles) than in primary tumors (gray circles) (p < 0.001) (Fig 7A). More careful analysis of those 28 cases in which we had patient-matched primary tumors and lymph node metastases revealed that VRK1 expression was higher in the lymph nodes than the primary tumor in 24 of the 28 cases (Fig 7B). These data strengthen the hypothesis that VRK1 affects later stages of tumor progression.

In addition, we used Oncomine’s online database to assess the upregulation of VRK1 mRNA in breast cancer. Upon analyzing the Curtis Breast database, which profiles the expression of >19,273 genes in >2,000 cases, VRK1 was in the top 5% (Fig 7C, boxes 2,3,6–9) or 10% (Fig 7C, boxes 5 and 11) of genes that were upregulated in multiple sub-types of invasive breast carcinoma relative to normal breast tissue. VRK1 was only in the top 25% of upregulated genes in ductal breast carcinoma *in situ* or mucinous breast carcinoma (boxes 4, and 10, respectively), and showed no significant upregulation in benign breast neoplasms (box 1). These data, coupled with our IHC data, suggest that VRK1 mRNA and protein levels are elevated during breast cancer progression.

To determine whether VRK1 overexpression is prognostic in breast cancer patients, we examined Kaplan-Meier ten year relapse-free survival curves for patients diagnosed with high-VRK1 expressing vs. low-VRK1-expressing cancers. High VRK1 expression showed a significant correlation with decreased relapse-free survival (Fig 7D). Interestingly, this prognostic correlation held true for patients with ER+ (Fig 7E) but not ER- breast cancer (Fig 7F). Cumulatively, the data shown in Fig 7 argue that VRK1 is frequently overexpressed in later stages of breast cancer (A-C) and that overexpression shows a statistically significant correlation with poor clinical outcome (7D, E).

## Discussion

### Impact of VRK1 overexpression on mammary epithelial cells in 2D and 3D culture

Although VRK1 has been considered a pro-proliferative kinase and overexpression is associated with a variety of cancers, it has been unclear how it might contribute to cancer initiation or progression. VRK1 overexpression does not affect cellular doubling time [this paper and [5]] or lead to the changes in cell morphology or monolayer appearance that are associated with transformation. The work described herein provides a new perspective by revealing that VRK1 overexpression augments the epithelial properties of cells in culture. We have shown that VRK1 overexpression reduces both single-cell and sheet migration (Figs 1 and 2, respectively), as well as invasion (S3 Fig) in 2D culture. These changes are accompanied by a downregulation of the EMT transcriptional repressors snail, slug, and twist1, and an upregulation of the epithelial cell-cell adhesion molecules E-cadherin and claudin1 (Fig 4). The stability of the snail, slug, and twist1 proteins is regulated by phosphorylation [43–45]. However, we found no differences in the t_1/2_ of the aforementioned proteins in VRK1-overexpressing vs. control cells (not shown). In contrast, we observed significant reductions in mRNA levels (Fig 4D) that were sufficient to account for the decreased protein levels of snail, slug, and twist1 in 3XF-VRK1-overexpressing cells. How VRK1 regulates the transcription of snail, slug, and twist1 is an area of future study, but it is plausible to speculate that VRK1 may phosphorylate upstream transcription factor(s).

Further evidence of the augmented epithelial properties induced by VRK1 overexpression comes from 3D matrigel culture experiments, in which cells proliferate and organize into spherical epithelial sheets surrounding hollow lumens (acini). VRK1-overexpressing cells display accelerated acinus growth (Fig 5). Cell cycle analysis showed that VRK1-overexpression increased cell proliferation at early days of 3D culture (Fig 6): at 4 days post-seeding, there were ten-fold more cells in the VRK1-overexpressing sample than in the controls. VRK1 overexpression may make cells more responsive to the growth-promoting signals from matrigel and/or enable cells to form an epithelial sheet more rapidly. Both of these parameters may contribute to accelerated acinus growth at early times.

In the original transcriptome analysis of 3D acini by Fournier et al. [27], endogenous VRK1 transcript levels declined by days 5–7 of culture. This shut-down, occurring at a time when acini cease proliferating and initiate maturation, is to be expected, since the VRK1 promoter is known to become repressed as cells enter G0 [46]. (The VRK1 protein has a long t_1/2_ (several days), and will therefore remain detectable after transcription ceases.) As shown in Fig 6D, the levels of overexpressed 3XF-VRK1 protein are always higher than the peak levels of endogenous VRK1; compare “vector” and “3XF-VRK1” lanes at each time point. Nevertheless, (Fig 6 B), the vast majority of cells at days 7 and 12 were in G0/G1 in all samples. These data indicate that although VRK1 overexpression promotes initial acinus growth, it does not override the transition to the later stages of acinar maturation, characterized by the halting of cell proliferation and the apoptosis of luminal cells.

Interestingly, the effect of VRK1 overexpression on 3D structures is similar to the phenotype induced by Akt activation, which leads to enhanced cell proliferation in early morphogenesis, but is not sufficient to overcome proliferative suppression in late morphogenesis [47]. Activated Akt can cooperate with other oncogenes such as cyclin D1 and HPV E7, amplifying the proliferation that they induce even in the absence of exogenously added growth factors [47]. Examining the cooperative potential of VRK1 overexpression with other oncogenes may be of interest for future study.

### How might VRK1-induced MET contribute to breast cancer progression?

**Fig 8:**
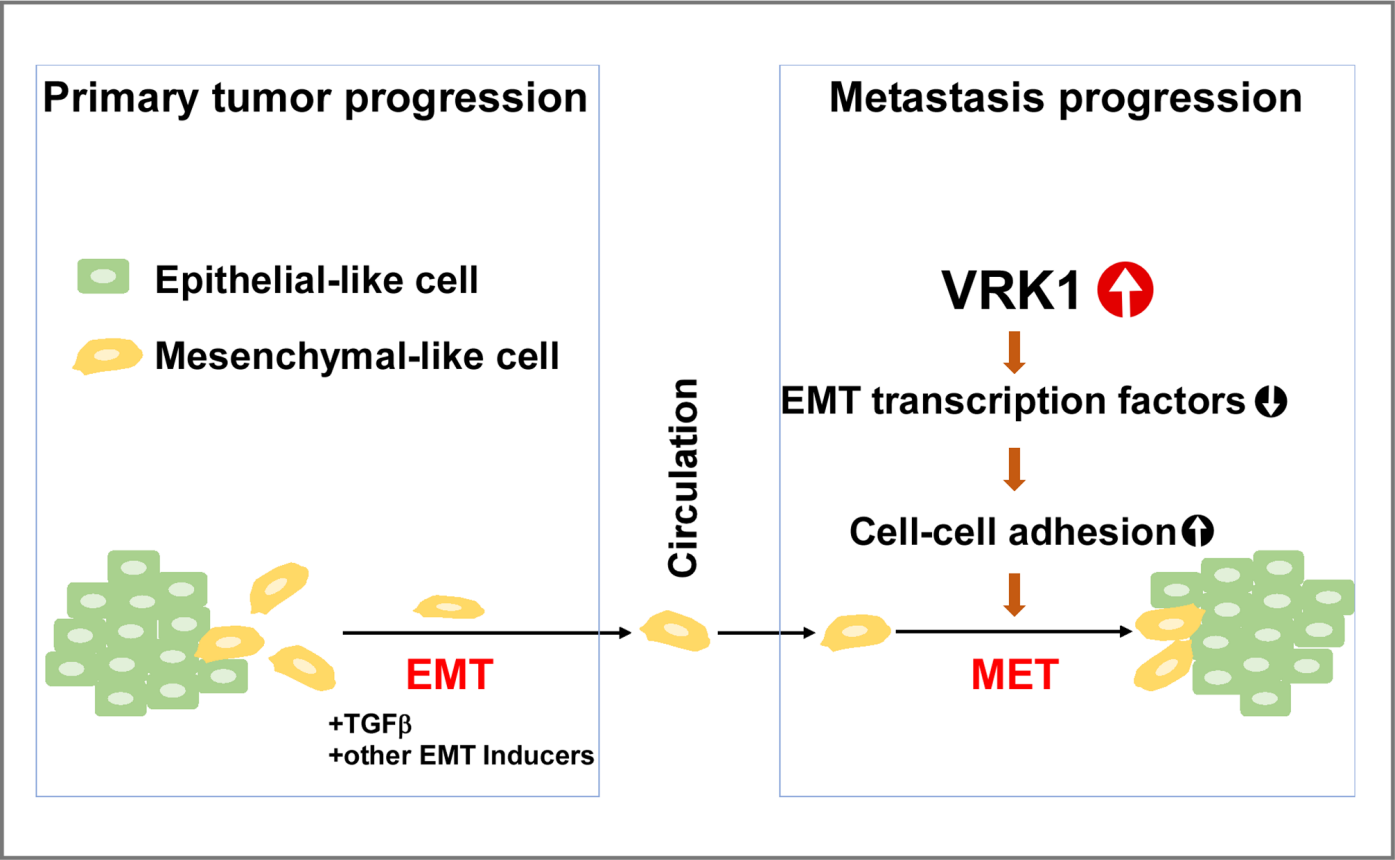
Model for VRK1’s role in breast cancer progression. During primary tumor progression, mammary epithelial cells lose cell-cell junctions and acquire a mesenchymal phenotype, a process known as epithelial-to-mesenchymal transition (EMT). These mesenchymal-like cells are thought to be more motile, less proliferative, and are competent to intravasate into and extravasate out of the blood stream. After extravasation, we propose that VRK1 promotes cancer cell colonization by inducing a partial mesenchymal-to-epithelial transition (MET) by downregulating the expression of mesenchymal markers (snail, slug and twist1 transcriptional repressors, as well as vimentin) and upregulating the expression of epithelial markers (E-cadherin and claudin1).

Our cell culture experiments provide conclusive evidence that VRK1 augments the epithelial properties of MCF10a and MDA-MB-231 cells in 2D culture, and accelerates acinus formation by MCF10a cells in 3D culture. How might the induction of MET by VRK1 overexpression be relevant to breast cancer development or progression *in vivo*? Two opposing hypotheses would be that either overexpression of VRK1 might retard the shedding of metastatic cells from the primary tumor or conversely, it might enhance the colonization of distal sites by metastatic cells. Our IHC analyses of TMAs indicate that VRK1 protein levels are increased in lymph node metastases as compared to patient-matched primary mammary tumors (Fig 7). When considered in conjunction with data mined from public databases (to be discussed below), we propose that the MET transition seen upon VRK1 overexpression might be associated with breast cancer progression by facilitating the colonization of metastatic cells as depicted in Fig 8. Certainly, future work aimed at testing this hypothesis directly in animal models is needed before its validity and relevance to human cancer can be assessed.

During metastasis, while EMT is crucial for tumor invasion and dissemination through the loss of E-cadherin, the opposite process, the mesenchymal-epithelial transition (MET), allows cancer cells to colonize successfully at secondary sites through the re-expression of epithelial genes [30, 31]. Several lines of evidence indicate that cells at distant metastatic sites display epithelial morphology, and display elevated E-cadherin expression relative to the cells of the primary tumor [32]. For example, forced expression of miR-200, which downregulates the ZEB1 transcriptional repressor and upregulates E-cadherin, led to the formation of 4T07 metastases in the lung and liver [33]. Furthermore, upregulation of E-cadherin in human prostate cancer PC-3/S cells enhanced their tumorigenicity [34]. In a reversible EMT model, expression of twist1 induced EMT and subsequent repression of twist1 led to MET. Inactivation of twist1 was required for the formation of distant metastases in a murine squamous cell carcinoma model [35].

### Association of VRK1 overexpression with breast cancer sub-types and correlation with clinical prognosis: Mining of public datasets

To complement our own IHC analysis of VRK1 overexpression in breast cancer patients (Fig 7A and 7B), we took advantage of publically available repositories of transcriptomic and patient outcome data. Oncomine (www.oncomine.org) allowed us to mine transcriptomic data from the large Curtis breast cancer dataset [>2,000 patients] [48]. Comparing normal breast tissue to tumors of all sub-types within this dataset, we determined that VRK1 ranked within the top 5% or 10% of overexpressed genes in the majority of invasive breast cancer subtypes (Fig 7C). It is worth putting this analysis within the context of previous data. In 2006, Fournier et al. [27] identified a signature of 22 genes that were downregulated during acinus formation in two independent mammary cell lines; VRK1 was among these 22 genes. In 2008, Martin et al. assessed the same signature for its ability to predict clinical outcome using three independent breast cancer microarray datasets (Van de Vijver [295 patients], Wang [286 patients], Sorlie [118 patients]) [28]. The expression level of the signature as a whole was found to have prognostic accuracy for relapse-free survival. Individual genes within the signature were also tested for prognostic ability. Levels of VRK1 expression were prognostic for both the Wang (p = 0.0001; 9 of the 22 genes were prognostic) and Sorlie datasets (p = 0.0057; 7 of the 22 genes were prognostic), but not for the Van de Vijver dataset.

Taking this one step further, Finetti et al. (2008) [29] focused on the expression of protein kinase genes, and defined a kinome signature (16 kinases) that could differentiate between basal (high kinome expression) and luminal A (low kinome expression) breast cancers, and furthermore could stratify luminal A breast cancers into Aa (lowest kinome expression) and Ab (higher kinome expression) sub-types. VRK1 was one of the 16 kinases within this kinome signature. Kaplan-Meier relapse-free survival analysis of the 80 breast cancers studied by Finetti revealed that patients with luminal Aa tumors had a significantly better prognosis than those with luminal Ab or basal tumors. The authors then assessed expression levels of the kinome signature in three other published data sets (Wang, Loi and Van de Vijver) and again reported that patients with low-kinome expressing tumors (luminal Aa) had a better clinical prognosis than those with higher kinome expression (luminal Ab and basal).

To provide an updated assessment of the correlation between VRK1 overexpression and clinical prognosis, we utilized http://kmplot.com/analysis, which integrates gene expression and clinical data and is updated biannually. As shown in Fig 7D, high levels of VRK1 expression showed a significant correlation with reduced 10-year relapse-free survival (3,951 patients, hazard ratio 1.72, P<1E-16). Interestingly, further analysis indicated that the highly significant correlation between VRK1 overexpression and poor clinical outcome was only seen for ER+, and not ER- tumors.

Why might the impact of VRK1 overexpression on relapse-free survival hold true only for patients with ER+ [Fig 7 and [28]] and luminal Ab tumors [29]? It is possible that ER signaling has a direct impact on how VRK1 regulates the cell biological properties of epithelial cells. Given that VRK1 is expressed in all proliferative cell types, and appears to modulate fundamental cell biological properties, we think that this explanation is unlikely. Alternatively, the influence of any gene product on cancer progression may well depend on the larger network of signaling proteins that determine the behavioral profile of the cells in question. Undoubtedly, the clinical differences between ER+ vs. ER- tumors are multifactorial, with different genes and proteins contributing to tumor cell properties and interactions with the tumor microenvironment and immune system. Whatever influence VRK1 (or any other protein) might have on ER- tumor cells, no impact on relapse-free survival will be observed if the influence does not affect a rate-limiting step or push cells past a crucial barrier.

Taken together, our new insights into the influence of VRK1 on epithelial cell biology, and our assessment of VRK1 overexpression in patient samples, set the stage for future mechanistic studies into how VRK1 may modulate breast cancer progression.

## Supporting information

S1 Table RT-PCR primers. This table displays the sequences of the primers used in the real-time quantitative PCR (RT-PCR) experiments.
(XLSX)

S1 Fig Characterization of cell lines overexpressing or depleted of VRK1. **(A)** A representative immunoblot of MDA-MB-231 cells modified to stably express 3XF-VRK1 as well as the empty vector control cells. Black diamond indicates the endogenous VRK1 expression and triangle indicates 3XFLAG-tagged VRK1. **(B)** A representative immunoblot of MDA-MB-231 cells stably depleted of VRK1 using the lentiviral-mediated delivery of shVRK1-01 and shVRK1-02. Black diamond indicates endogenous VRK1. **(C)** A representative immunoblot of MCF10a cells stably depleted of VRK1 using the lentiviral-mediated delivery of shVRK1-01 and shVRK1-02. Black diamond indicates endogenous VRK1.
(PDF)

S2 Fig VRK1 overexpression does not affect cell-extracellular matrix (ECM) substrate adhesion in 2D culture. **(A)** Cell adhesion assay using crystal violet: cells were allowed to attach to the plates for 20 min at 37°C before removing the unattached cells. Attached cells were stained with crystal violet, and intensity was measured at 590 nm. **(B)** Cell adhesion assay using Calcein/AM dye. Cells pre-incubated with a Calcein/AM dye were allowed to attach to the matrix for 20 min at 37°C before removing the unattached cells. Attached cells were measured by assessing the fluorescent intensity. Means and standard error are plotted (n = 4).
(PDF)

S3 Fig VRK1 overexpression impairs cell invasion. **(A)** Serum-starved cells were added to the upper chamber of matrigel-coated transwell chambers. Lower chambers contained complete medium as a chemoattractant; cells were incubated at 37°C for 16h. Representative images of the underside of the filter containing DAPI-stained, invaded cells are shown. Scale bar = 100μm. **(B)** Quantification of percent invasion (normalized to appropriate vector control for each cell type) is shown (***p<0.001) (n = 3).
(PDF)

S1 Movie Live imaging microscopy of wound closure by stably-transduced MCF10A cells expressing empty vector. Confluent monolayers of cells were wounded, and wound closure was monitored by performing live imaging microscopy. Images were taken at 10X magnification every 30min for 18h.
(AVI)

S2 Movie Live imaging microscopy of wound closure by stably-transduced MCF10A cells expressing 3XF-VRK1. Confluent monolayers of cells were wounded, and wound closure was monitored by performing live imaging microscopy. Images were taken at 10X magnification every 30min for 18h.
(AVI)

S3 Movie Live imaging microscopy of wound closure by stably-transduced MCF10A cells expressing 3XF-VRK1^D177A^. Confluent monolayers of cells were wounded, and wound closure was monitored by performing live imaging microscopy. Images were taken at 10X magnification every 30min for 18h.
(AVI)
