# Correction: Overexpression of the VRK1 kinase, which is associated with breast cancer, induces a mesenchymal to epithelial transition in mammary epithelial cells

**DOI:** 10.1371/journal.pone.0307693

**Published:** 2024-07-18

**Authors:** Aye M. Mon, A. Craig MacKinnon Jr., Paula Traktman

The two images in the bottom row of Fig 2A are incorrect. Please see the correct Fig 2 here.

**Fig 2:**
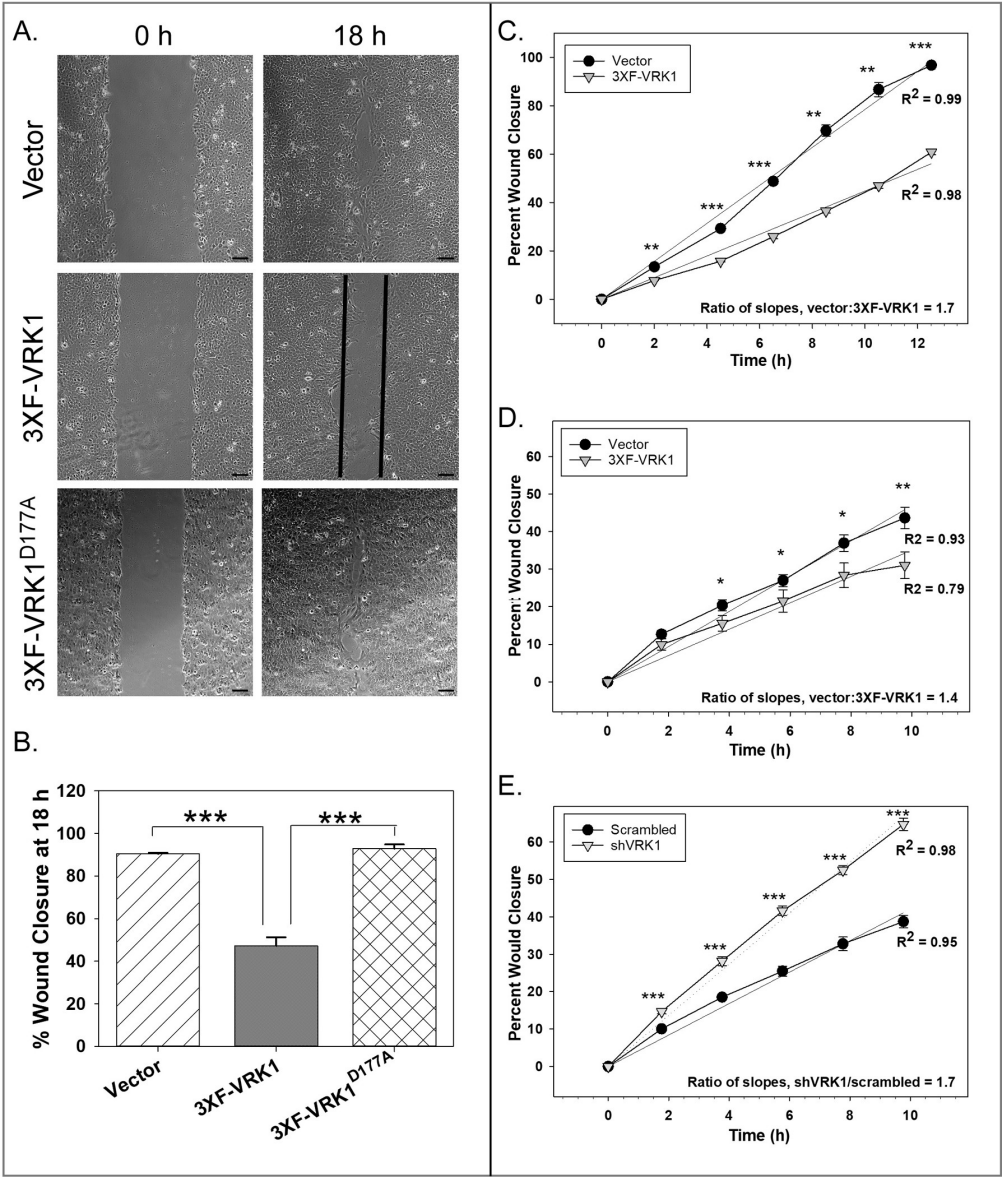
VRK1 overexpression significantly impairs epithelial sheet migration. **(A and B)** Confluent monolayers of the cell lines shown were wounded, and wound closure was monitored for 18h by live imaging microscopy. **A:** Representative images of vector control, 3XF-VRK1-overexpressing and 3XF-VRK1^D177A^-overexpressing cells are shown at 0 and 18h after wounding. Scale bar = 100μm. **B:** Quantification of the percent wound closure at 18 h (Mean and standard error are shown; ***p<0.001) (n = 4 images) **(C-E)** Data from representative IncuCyte wound scratch assays are shown. Using the IncuCyte live imaging system, the rate of wound closure was compared pairwise for vector control MCF10A cells (black circles) and 3XF-VRK1 overexpressing MCF10A cells (gray triangles) **(C)**, or for vector control MDA-MB-231 cells (black circles) vs. 3XF-VRK1- overexpressing MDA-MB-231 cells (gray triangle) **(D)**, or for scrambled shRNA control MDA-MB-231 cells (black circles) vs. VRK1-depleted MDA-MB-231 cells (gray triangles) **(E)**. Slope ratios were determined after linear regression analysis. Significance was assessed using a Student’s *t-test* for each time point (mean and standard error are shown; *p ≤ 0.05; **p ≤ 0.01;***p ≤ 0.001) (n = 6 images).
