# Supplementary figures and images for: Overexpression of the VRK1 kinase, which is associated with breast cancer, induces a mesenchymal to epithelial transition in mammary epithelial cells
Source: PLoS One. 2018 Sep 4;13(9):e0203397. doi: 10.1371/journal.pone.0203397 (PMC6122820; doi:10.1371/journal.pone.0203397)

Figure S1

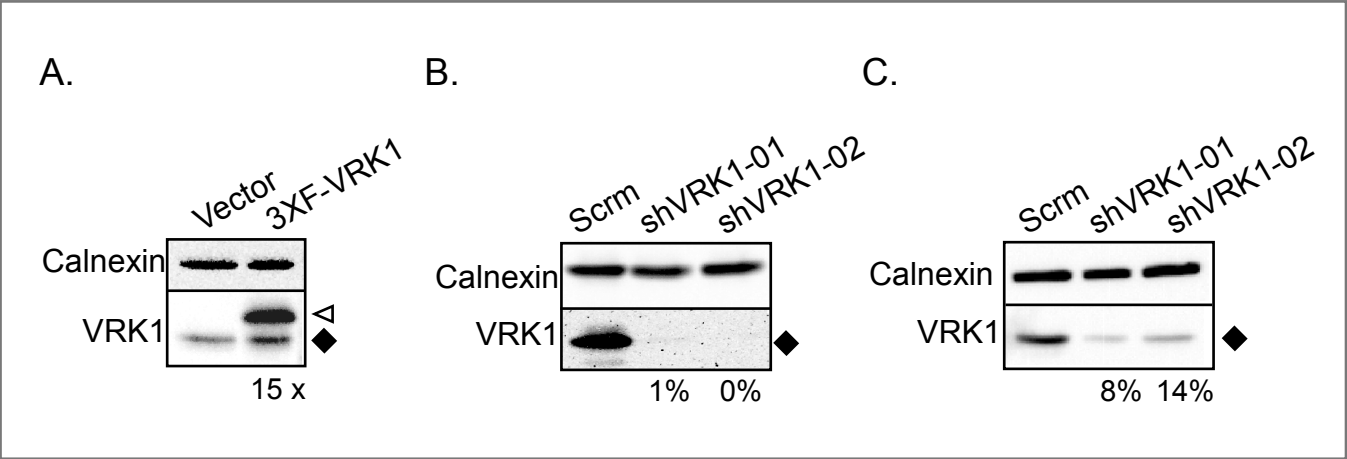

Supplement: S1 Fig — (A) A representative immunoblot of MDA-MB-231 cells modified to stably express 3XF-VRK1 as well as the empty vector control cells. Black diamond indicates the endogenous VRK1 expression and triangle indicates 3XFLAG-tagged VRK1. (B) A representative immunoblot of MDA-MB-231 cells stably depleted of VRK1 using the lentiviral-mediated delivery of shVRK1-01 and shVRK1-02. Black diamond indicates endogenous VRK1. (C) A representative immunoblot of MCF10a cells stably depleted of VRK1 using the lentiviral-mediated delivery of shVRK1-01 and shVRK1-02. Black diamond indicates endogenous VRK1. (PDF) [file pone.0203397.s002.pdf]

Figure S2

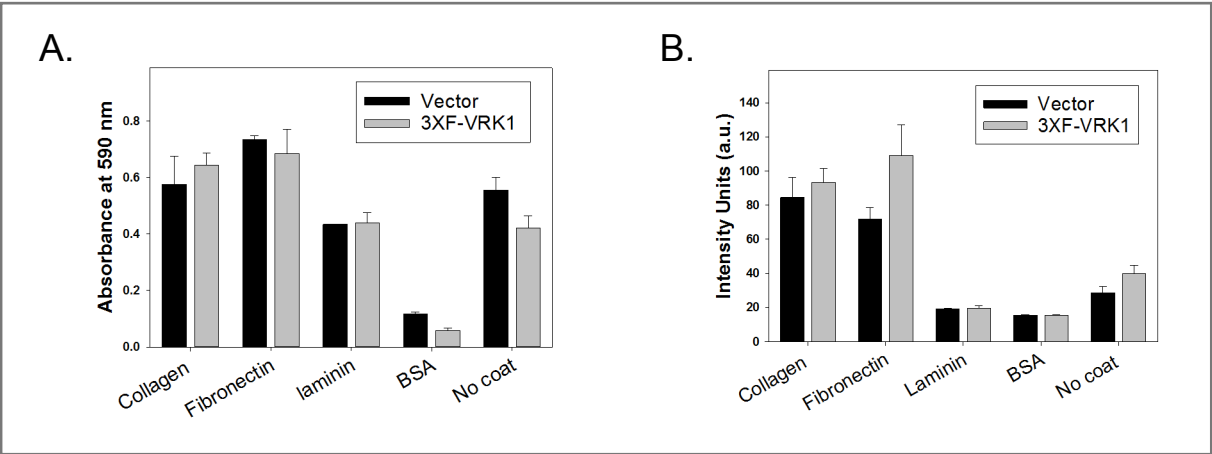

Supplement: S2 Fig — (A) Cell adhesion assay using crystal violet: cells were allowed to attach to the plates for 20 min at 37°C before removing the unattached cells. Attached cells were stained with crystal violet, and intensity was measured at 590 nm. (B) Cell adhesion assay using Calcein/AM dye. Cells pre-incubated with a Calcein/AM dye were allowed to attach to the matrix for 20 min at 37°C before removing the unattached cells. Attached cells were measured by assessing the fluorescent intensity. Means and standard error are plotted (n = 4). (PDF) [file pone.0203397.s003.pdf]

Figure S3

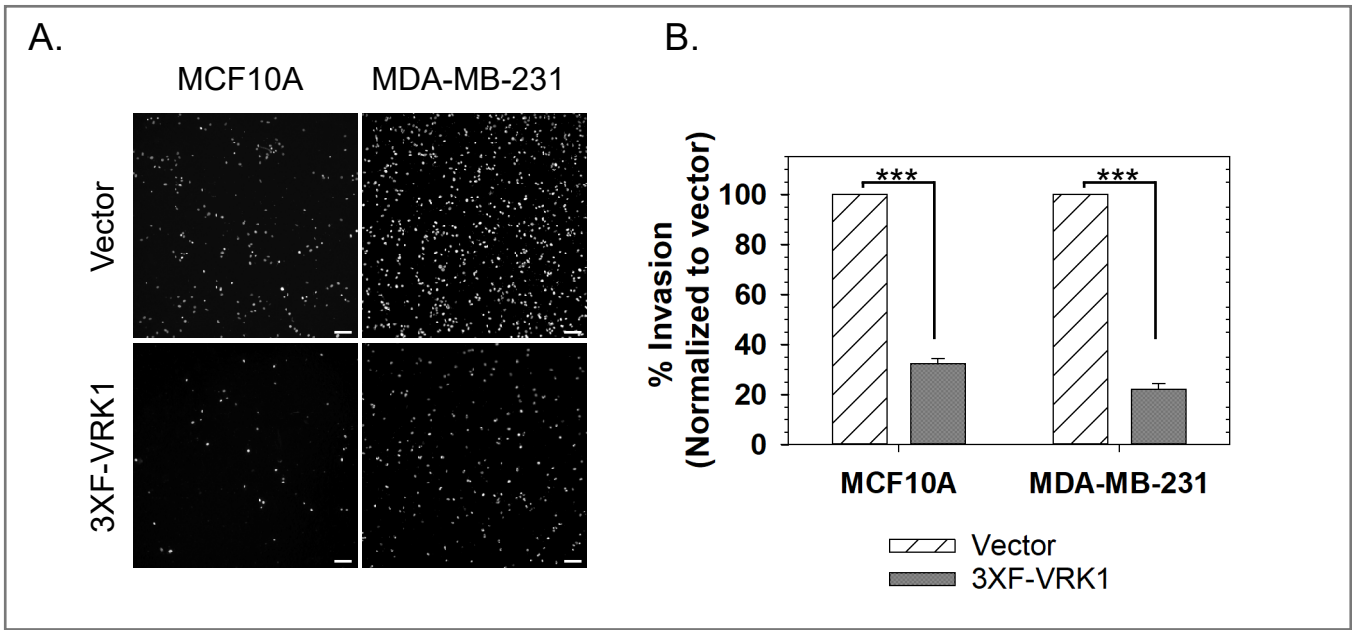

Supplement: S3 Fig — (A) Serum-starved cells were added to the upper chamber of matrigel-coated transwell chambers. Lower chambers contained complete medium as a chemoattractant; cells were incubated at 37°C for 16h. Representative images of the underside of the filter containing DAPI-stained, invaded cells are shown. Scale bar = 100μm. (B) Quantification of percent invasion (normalized to appropriate vector control for each cell type) is shown (***p<0.001) (n = 3). (PDF) [file pone.0203397.s004.pdf]
